# A Liquid Crystal Elastomer‐Based Unprecedented Two‐Way Shape‐Memory Aerogel

**DOI:** 10.1002/advs.202102674

**Published:** 2021-09-26

**Authors:** Meng Wang, Ying Song, Hari Krishna Bisoyi, Jian‐Feng Yang, Li Liu, Hong Yang, Quan Li

**Affiliations:** ^1^ Institute of Advanced Materials School of Chemistry and Chemical Engineering and Jiangsu Hi‐Tech Key Laboratory for Biomedical Research Southeast University Nanjing 211189 China; ^2^ Advanced Materials and Liquid Crystal Institute and Chemical Physics Interdisciplinary Program Kent State University Kent OH 44242 USA

**Keywords:** aerogels, liquid crystal elastomers, shape‐morphing materials, two‐way shape‐memory aerogels

## Abstract

With the advantage of reversible shape‐morphing between two different permanent shapes under external stimuli, the two‐way shape‐memory aerogel is expected to become a preferred aerogel for developing practical applications in actuators, sensors, robotics, and more. Herein, the first two‐way shape‐memory liquid crystal elastomer (LCE)‐based aerogel is prepared by an orthogonal heat and light curing strategy coupled with an intermediate mechanical stretching step. The differential scanning calorimetry, temperature‐varied wide‐angle X‐ray scattering, and polarizing optical microscope results indicate that the aerogel possesses a liquid crystal phase and the insider mesogens are well‐oriented along the stretching direction. In addition to having superior compressibility and excellent shape stability, this LCE‐based aerogel can perform a reversible shape deformation during the heating/cooling cycles with a shrinkage ratio of 37%. The work, that is disclosed here, realizes a truly two‐way shape‐memory behavior rather than the one‐way shape deformation of traditional polymer aerogel materials, and may promote potential applications of this novel LCE‐based aerogel material in control devices, soft actuators, and beyond.

## Graphical abstract

The first two‐way shape‐memory liquid crystal elastomer (LCE)‐based aerogel, possessing both the porous structure of typical polymeric aerogel materials and the reversible shape morphing of the LCEs, is prepared by an orthogonal heat and light curing strategy coupled with an intermediate mechanical stretching step.

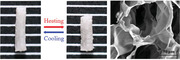

## 1 Introduction

Aerogels, formed by replacing the liquid components in the gel pores with gases, are recognized as the lightest solid materials in the world.^[^ ^1^ ^]^ As a most promising porous material, aerogels have unique properties such as low density, low thermal conductivity, and large surface area, which endow aerogels with versatile applications in catalyst supports, sensors, absorbents, aerospace materials, and so on. In particular, polymeric aerogels, derived entirely from polymeric materials, exhibit the outstanding advantages including good mechanical properties and environmental stability, which render them suitable for a wide range of potential applications.^[^ ^2^, ^3^, ^4^, ^5^, ^6^, ^7^ ^]^ As a kind of distinctive polymeric aerogels, shape‐memory polymeric aerogels (SMPAs) have attracted much attention in recent years owing to their porous structures as well as the capability of shape recovery triggered by external stimuli. Since Rowan reported the first thermal‐responsive SMPA material with the capabilities of shape fixation and shape recovery in 2016,^[^ ^8^ ^]^ many research groups have focused on this field and reported a series of novel SMPAs originating from traditional shape memory polymeric materials.^[^ ^9^, ^10^, ^11^, ^12^ ^]^

**Figure 1:**
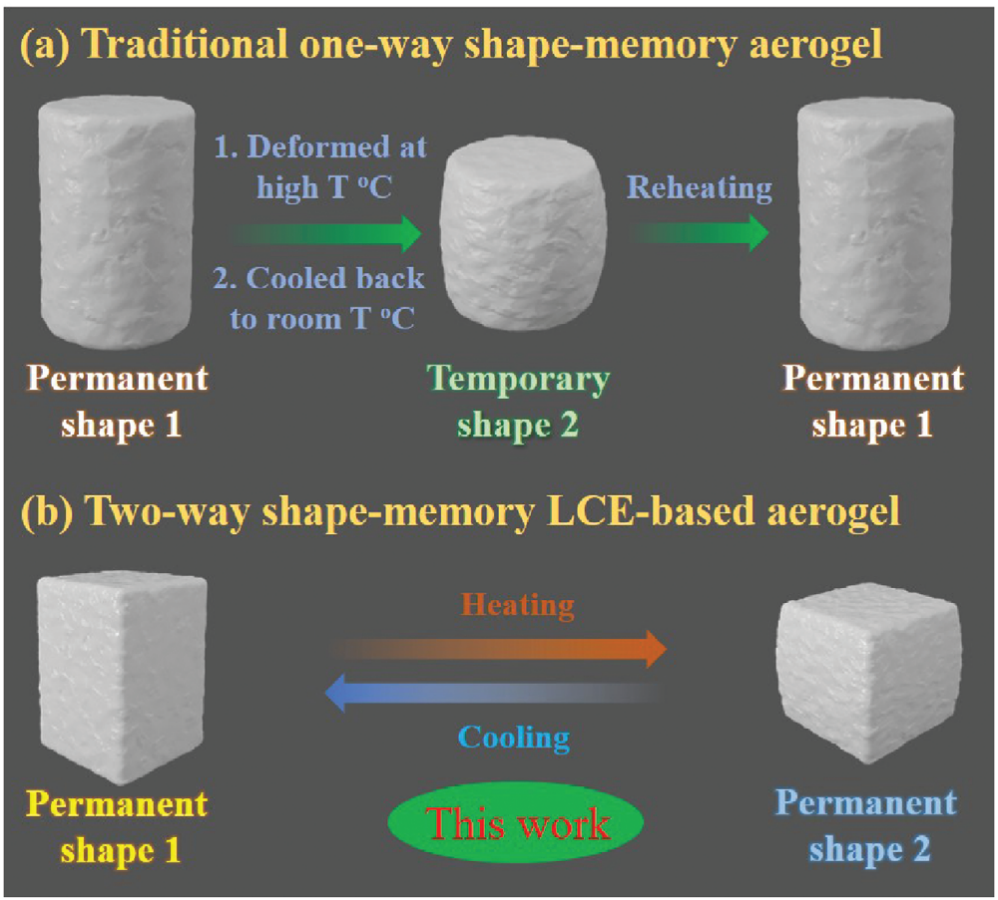
a) Traditional one‐way shape‐memory polymeric aerogel. b) Two‐way shape‐memory LCE‐based aerogel designed in this work.

A significant commonality of these previous reported SMPA materials is that they can only perform a one‐way shape‐memory deformation. As shown in **Figure** **1**a, the one‐way SMPAs (1W‐SMPA, permanent shape 1) were first deformed at a relatively high temperature (usually higher than their glass transition or melting temperatures) and then cooled back to room temperature to obtain a temporary shape (temporary shape 2). After reheating, 1W‐SMPA could relax from the temporary shape (temporary shape 2) to the permanent shape (permanent shape 1). However, this shape deformation from the temporary shape 2 to the permanent shape 1 was a nonreversible process, and the aerogel needed to be deformed again to obtain a new temporary shape to repeat the one‐way shape‐memory behavior. Obviously, the impermanence and non‐reversibility of the one‐way shape deformation have become major obstacles to the practical applications of 1W‐SMPAs. On the other hand, two‐way SMPAs (2W‐SMPAs) have the special advantage of reversible shape‐morphing between two different permanent shapes under external stimuli in comparison with 1W‐SMPAs, and are promising to be the preferred aerogels for practical applications in actuators, sensors, robotics, aerospace technology, and more. However, to the best of our knowledge, 2W‐SMPAs have not been reported to date.^[^ ^13^, ^14^ ^]^ So, the design, preparation and characterization of 2W‐SMPAs with reversible shape‐morphing capability and porous structures are all new challenges. Herein, we present the first 2W‐SMPA sample, for which reversible shape deformation from a permanent shape (permanent shape 1) to the other permanent shape (permanent shape 2) under stimuli has been realized (Figure 1b).

It is a natural and feasible strategy to design and fabricate 2W‐SMPAs starting from traditional two‐way shape‐memory polymers. As an intriguing category of two‐way shape‐memory materials, liquid crystal elastomers (LCEs),^[^ ^15^, ^16^, ^17^, ^18^ ^]^ possessing both anisotropic properties of liquid crystals (LCs) and the rubber elasticity, could perform a large and reversible macroscopic shape deformation between two different permanent shapes due to the change in the molecular order of the mesogens during the LC‐to‐isotropic phase transition process induced by external stimuli, and have exhibited prosperous applications in mechanical actuators,^[^ ^19^, ^20^, ^21^, ^22^ ^]^ artificial organs,^[^ ^23^, ^24^ ^]^ smart surfaces,^[^ ^25^, ^26^ ^]^ and more. Such excellent properties suggested the potential of LCEs to be suitable materials for realizing novel 2W‐SMPAs. In a traditional LCEs fabrication process, a pre‐crosslinked LCE with lightly crosslinked networks was formed in the first stage, followed by shape programming and second crosslinking step to further lock the anisotropic network to obtain a new permanent shape.^[^ ^27^ ^]^ However, it is paradoxical that the pre‐crosslinking stage of classical LCE materials only needed several hours, but the preparation process of traditional polymeric aerogels,^[^ ^28^ ^]^ including sol‐gel transition, network perfection (aging), and gel–aerogel transition, always took several days, and was significantly longer than the pre‐crosslinking time of classical LCE materials. Such a long reaction time would lead to the complete crosslinking of the LCE‐based aerogel before the shape programming process, which meant that there would be no excess reactive groups to fix the new programmed shape in the second crosslinking stage.

To resolve this competing issue, we herein adopt an orthogonal thermal and photo curing strategy coupled with an intermediate mechanical stretching step to fabricate the first LCE‐based 2W‐SMPA material, with the goal of accessing an aerogel that could possess both porous structures and reversible shape‐morphing ability. The two non‐interfering crosslinking reactions, including thermal‐induced thiol‐ene click chemistry^[^ ^29^ ^]^ and ultraviolet (UV)‐triggered H‐atom abstraction reaction,^[^ ^30^, ^31^ ^]^ respectively occurred in the first and second crosslinking stages. In the first stage, the classical polymer aerogel preparation process was performed to give a polydomain LCE‐based aerogel coupled with a facile thermally‐induced thiol‐ene click chemistry method and a following supercritical CO_2_ extraction technology. In the second stage, the polydomain LCE‐based aerogel sample was first uniaxially stretched to force the mesogens to orient along the longitudinal axis. Then, UV‐light was used to illuminate the aerogel to initiate the H‐atom abstraction reaction between the benzophenone groups and C—H bonds from adjacent carbon chain to lock the uniaxial orientation of mesogens, and further to give an oriented LCE‐based aerogel. These two non‐interfering curing reactions designed in this work not only ensured the formation of porous structures of the aerogel, but also enabled that after a long period of the aerogel preparation and shape programming processes, there were still new crosslinking reaction sites to fix the new shaped LCE‐based aerogel. The mesomorphic properties, porous structures, mechanical properties, and thermal‐induced two‐way shape‐memory behavior of the prepared LCE‐based aerogel were investigated in this work.

## 2 Results and Discussion

**Figure 2:**
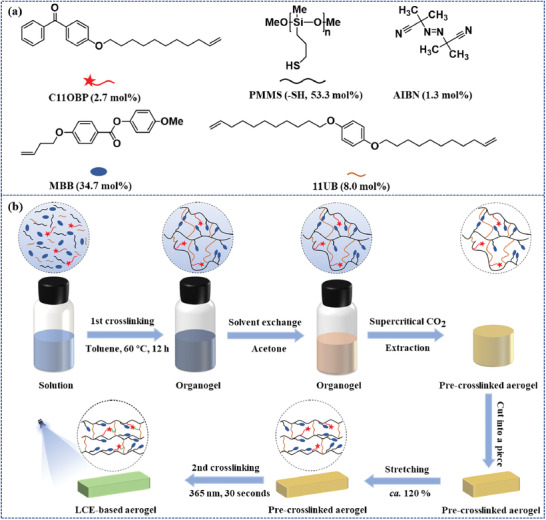
a) Chemical structures of PMMS, MBB, 11UB, C11OBP, and AIBN. b) Schematic illustration of the fabrication process of the uniaxially stretched LCE‐based aerogel sample.

All the preparation protocols and instrumentation descriptions regarding the preparation of the uniaxially‐stretched LCE‐based aerogel sample, detailed in the Supporting Information and schematically illustrated in **Figure** **2**, are summarized below. In this work, we selected polysiloxane LCE system because it possessed highly flexible backbone, good mechanical stability, low glass transition temperature (*T*_g_) and relatively low LC‐to‐isotropic phase transition temperature (*T*_iso_), and so on. As shown in Figure 2b; Figure S7, Supporting Information, in the first crosslinking stage, the poly‐[3‐mercaptopropylmethylsiloxane] (PMMS), the vinyl‐terminated end‐on mesogen 4‐methoxyphenyl‐4‐(1‐buteneoxy) benzoate (MBB), the di‐vinyl‐terminated crosslinker 1,4‐bis‐undec‐10‐enyloxy‐benzene (11UB), another benzophenone‐terminated crosslinker 4‐(10‐undecenyloxy) benzophenone (C11OBP), and the initiator azodiisobutyronitrile (AIBN) were dissolved in toluene and then transferred into a sealed transparent glass bottle. After ultrasonication, the mixture was heated to 60 °C for 12 h to carry out the thermal‐induced thiol‐ene click polymerization to produce a transparent organogel. The transparent organogel was then completely soaked in acetone, and the solvent was exchanged by fresh acetone for three times to remove excess toluene and impurities from the organogel, to give an opaque milky‐white organogel. After supercritical CO_2_ extraction process, the organogel was dried to obtain the polydomain LCE‐based aerogel. The aerogel sample was further cut into a piece and uniaxially‐stretched to realize an ordered arrangement of the mesogens in the LCE strands of the aerogel. The uniaxially‐stretched aerogel was exposed to the UV‐light to perform the photo‐induced H‐atom abstraction reaction between the benzophenone groups and C—H bonds from the adjacent carbon chain to fix the uniaxial orientation and complete the second crosslinking stage, thereby forming an LCE‐based aerogel with oriented mesogens.^[^ ^30^, ^31^ ^]^ The thiol‐ene click chemistry performed in the first stage only required cheap radical initiators as catalysts, which could be easily removed during the following solvent exchange process. Toluene was selected as the reaction solvent because its boiling point temperature (110.6 °C) was higher than the reaction temperature (60 °C). During the reaction process, toluene hardly vaporized in the sealed bottle, which was conducive to maintaining the microstructure and macroscopic shape of the organogel. Besides, toluene and acetone were mutually soluble, which was beneficial for the subsequent solvent exchange and supercritical CO_2_ extraction processes. Benzophenone‐terminated crosslinker C11OBP was involved in the second crosslinking stage to fix the deformed aerogel because benzophenone was chemically inert in the absence of light and would not be affected by the lengthy aerogel preparation process during the first crosslinking stage.

**Figure 3:**
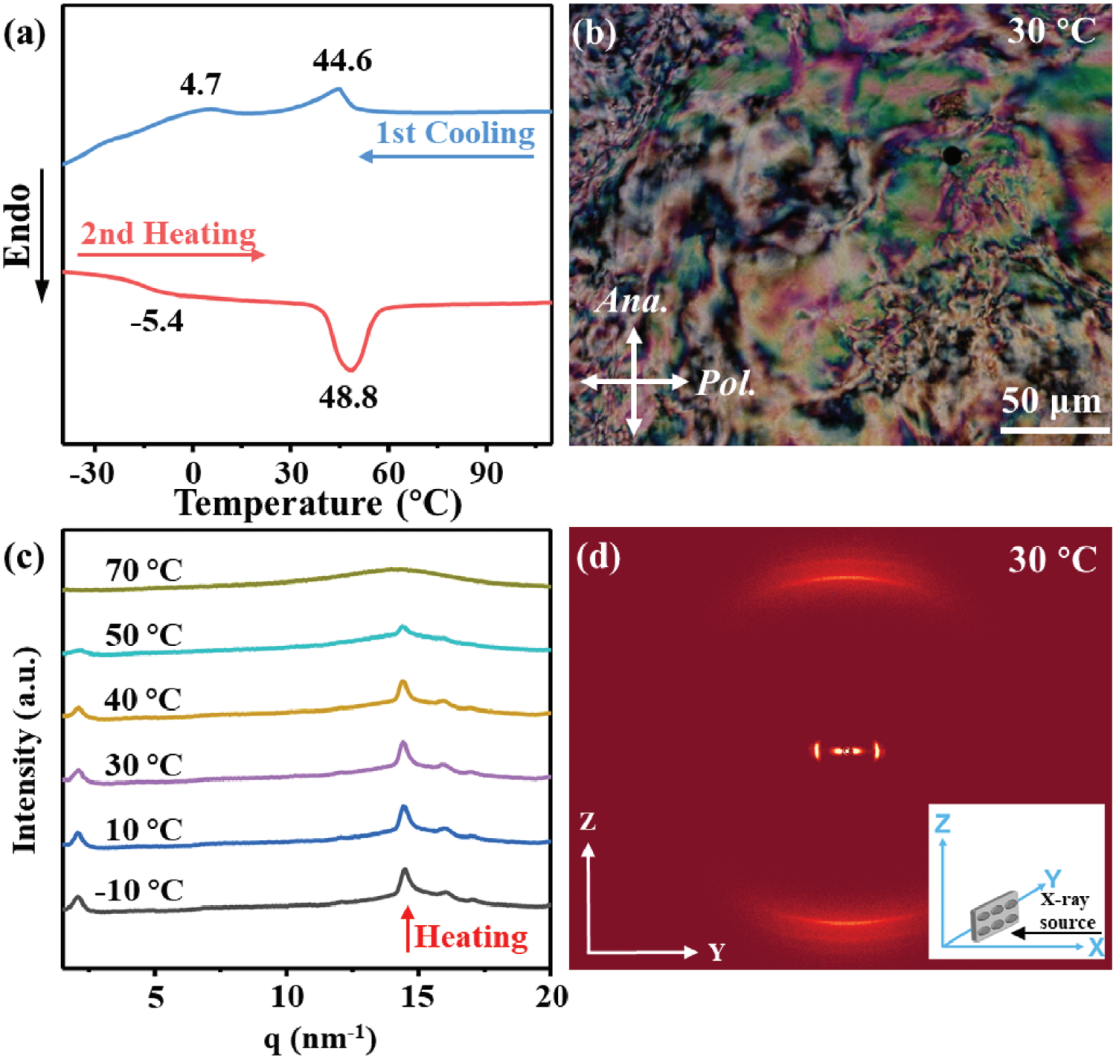
a) DSC curves of LCE‐based aerogel sample during the first cooling and second heating scans at a rate of 10 °C min^−1^ under nitrogen atmosphere. b) POM image of the LCE‐based aerogel sample at 30 °C. c) 1D‐WAXS patterns of the LCE‐based aerogel sample during the heating process. d) 2D‐WAXS pattern of the oriented LCE‐based aerogel sample at 30 °C.

**Figure 4:**
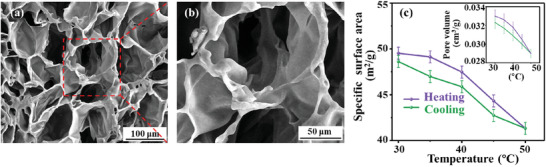
a,b) SEM images of the LCE‐based aerogel sample. c) The specific surface area and pore volume of the LCE‐based aerogel sample plotted as a function of temperature.

The thermal and mesomorphic properties of the LCE‐based aerogel with oriented mesogens were investigated by differential scanning calorimetry (DSC), temperature‐varied wide‐angle X‐ray scattering (WAXS), and polarized optical microscope (POM). As shown in **Figure** **3**; Figures S9–S11, Supporting Information, the LCE‐based aerogel possesses a LC phase in the temperature region of ≈ −5.4–48.8 °C and the mesogens in the aerogel strands were well aligned along the stretching direction. The field emission scanning electron microscopy (SEM) technique was used to observe the internal microscopic morphologies and skeleton structure of the LCE‐based aerogel. As shown in **Figure** **4**a,b, the LCE‐based aerogel showed a distinct hierarchical porous structure, and the internal walls were smooth and no obvious wrinkles were observed on the pore walls, indicating that the skeleton structures did not collapse during the solvent exchange and supercritical CO_2_ drying process. The physical properties of the LCE‐based aerogel sample were measured and analyzed to evaluate the porosity of the sample at different temperatures. The bulk density *ρ*_b_ of the sample was calculated to be 0.217 ± 0.004 g cm^−3^, and the LCE strand skeleton density *ρ*_s_ measured by the automatic true density analyzer was 1.0417 ± 0.021 g cm^−3^. The sample porosity, calculated by the formula (1−*ρ*_b_/*ρ*_s_) × 100%, was 79.2 ± 0.1% at room temperature, indicating a porous structure of the LCE‐based aerogel. After degassing at three different temperatures (30 °C, 40 °C, 50 °C) for 8 h, the LCE‐based aerogel sample was instantly frozen by the liquid nitrogen to fix the shape, and then the specific surface area and pore volume were measured in a liquid nitrogen atmosphere. As illustrated in Figure 4c, the specific surface areas of the LCE‐based aerogel sample at 30 °C and 40 °C were basically similar in the range of ≈48–50 m^2^ g^−1^. Similarly, the pore volumes of the aerogel sample were ≈0.033 ± 0.001 g cm^−3^ at 30 °C and 40 °C. When the temperature reached 50 °C, obvious decreases of the specific surface area and pore volume were both recorded, implying that the sample shrunk or collapsed during the heating process. After cooling back to the room temperature, the values of specific surface area and pore volume returned close to the initial value (Figure 4c), indicting a shape recovery of this LCE‐based aerogel sample. These data preliminarily suggested that the LCE‐based aerogel sample might have performed a reversible shape deformation process in the heating/cooling cycle.

**Figure 5:**
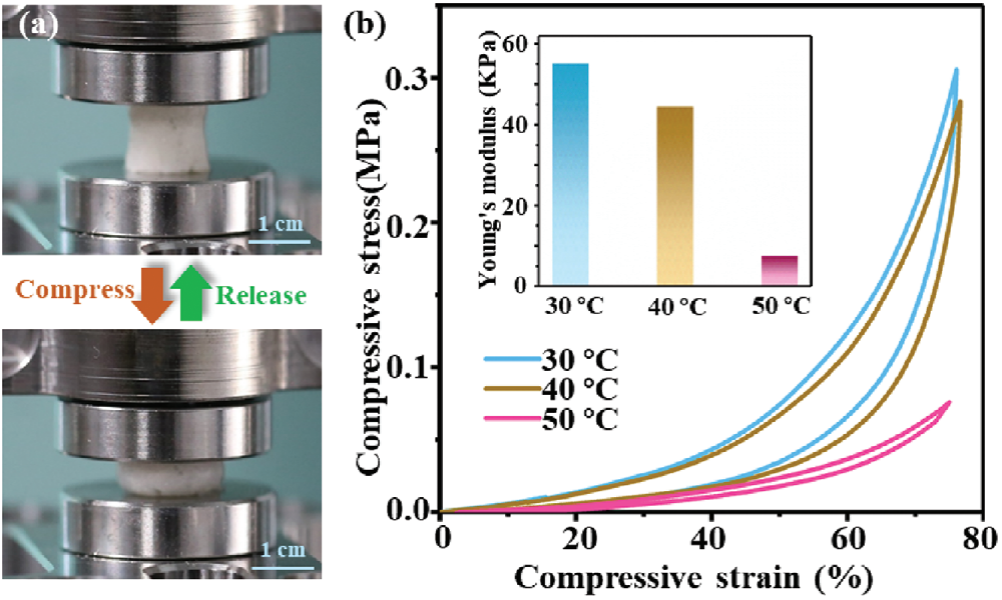
a) Compression and recovery test of the LCE‐based aerogel sample along the stretching direction. b) The cyclic compression–relaxation curves and the corresponding Young's modulus of the LCE‐based aerogel sample at different temperatures.

In addition to the porous structure, the mechanical properties and shape recovery ability of the LCE‐based aerogel were further investigated by the dynamic mechanical analyzer (DMA) through a series of compressive tests. As shown in **Figure** **5**a; Figure S14 and Videos S1 and S2, Supporting Information, after 75% compression along or perpendicular to the stretching direction, the LCE‐based aerogel sample could both quickly recover to the original shape with no significant damage, indicating that this aerogel had good compressibility and excellent shape stability. The uniaxial compression–decompression stress–strain diagrams of the LCE‐based aerogel sample at different temperatures (30 °C, 40 °C, 50 °C) were plotted in Figure 5b. The LCE‐based aerogel sample at three different temperatures showed remarkable toughness and flexibility under a 75% compression strain, owing to the soft PMMS skeleton, the moderate crosslinking density, and the large free space provided by the porous structure. Particularly, at 50 °C, the compression stress of the LCE‐based aerogel under a compression strain of 75% was 0.076 MPa, which was much lower than the compression stress of 0.3 MPa at 30 °C and 0.28 MPa at 40 °C, respectively. At 30 °C and 40 °C, the two compressive–stress curves of the aerogel sample both showed a slow increase before the 60% compressive strain and a significantly sharp increase after the 60% compressive strain. At the low compressive strain, the deformation of the LCE‐based aerogel mainly was the bending of internal walls. As the compression strain further increased above 60%, the aerogel became more and more compact, and the collision between adjacent walls promoted a rapid increase of resistance and compressive strength inside the LCE‐based aerogel. Correspondingly, as shown in Figure 5b, the LCE‐based aerogel sample exhibited Young's modulus of ≈50 KPa at 30 °C and 40 °C, and a relatively low value of 7 KPa at 50 °C, implying the softening or morphing of the LCE‐based aerogel when the temperature rose to above *T*_iso_.

**Figure 6:**
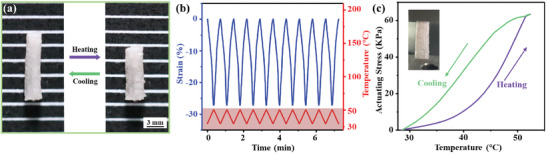
a) Thermal‐induced two‐way shape‐memory behavior of the LCE‐based aerogel sample. b) Representative strain (in isoforce mode) and the corresponding temperature diagram of the LCE‐based aerogel sample plotted against time. c) Representative isostrain (0.01%) measurement of the LCE‐based aerogel sample plotted against temperature.

Encouraged by the above data, the thermally‐induced two‐way shape‐memory behavior of the LCE‐based aerogel was further investigated by placing the long strip sample on top of a hot stage for heating/cooling cycles. As shown in **Figure** **6**a; Video S3, Supporting Information, the LCE‐based aerogel performed a conspicuously shrinking deformation along the stretching direction owing to the LC‐to‐isotropic phase transition. After cooling back to room temperature, the original shape was restored. During the heating–cooling process, the maximum shrinkage rate, defined as (*L*_0_ – *L*_iso_)/ L_iso_ (*L*_0_ was the longitudinal length of the LCE‐based aerogel measured at room temperature and *L*_iso_ was the shortest length of the LCE‐based aerogel measured at isotropic phase), was observed as 37%. DMA was further used to investigate the mechanical properties and shape recovery of this LCE‐based aerogel with the change of temperature. As schematically illustrated in Figure 6b, under the isoforce mode, the contraction strain of the LCE‐based aerogel sample, defined as (*L−L*_0_)/*L*_0_ (*L* is the measured length of LCE‐based aerogel sample at any temperature), changed from 0 to −27% during the heating process from 30 °C to 50 °C. Upon cooling, the strain value of the sample recovered to 0 from −27%. In addition, the strain of the LCE‐based aerogel changed reversibly during the next several heating and cooling processes, implying an excellent reversibility and repeatability of the LCE‐based aerogel. The actuating stress of the LCE‐based aerogel sample was further studied by using the isostrain mode of DMA where the LCE‐based aerogel sample was elongated by a constant 0.01% strain in tension. As illustrated in Figure 6c, the contractile force generated by the LCE‐based aerogel sample was measured as a function of the temperature. During the heating process, the LCE‐based aerogel sample produced corresponding actuating stress with variation of temperature, and the maximum stress value generated was 63 kPa. After cooling to room temperature, the actuating stress returned to the initial value of 0. The reversible change of the generated contractile force during the heating/cooling cycle confirmed that the LCE‐based aerogel sample possessed the two‐way shape‐memory ability.

## 3 Conclusion

A novel two‐way shape‐memory LCE‐based aerogel, possessing both the porous structure of typical polymeric aerogel materials and the reversible shape morphing of the LCEs, has been designed and prepared by an orthogonal thermal and photo curing strategy coupled with an intermediate mechanical stretching step. The DSC, POM, and WAXS results prove that the LCE‐based aerogel possesses a LC phase and the mesogens in the aerogel are well oriented along the stretching direction. Besides, the LCE‐based aerogel has distinct hierarchical porous structures, good compressibility and excellent shape stability, which are typical properties of conventional polymeric aerogels. Moreover, this LCE‐based aerogel could perform a reversible shrinking/expanding deformation during the heating/cooling cycle with a maximum shrinkage ratio of 37% and a generated contractile force of 63 kPa. In addition, the two‐way shape‐memory property of this LCE‐based aerogel possesses superior fatigue resistance during several heating/cooling cycles. To the best of our knowledge, this is the first example of polymer aerogel materials exhibiting two‐way shape‐memory behaviors under external stimuli, which might endow this novel LCE‐based aerogel material with potential applications in the field of control devices, soft actuators, and beyond.

## Conflict of Interest

The authors declare no conflict of interest.

## Supporting information

Supporting Information

Supplemental Video 1

Supplemental Video 2

Supplemental Video 3

## Data Availability

Data available in the Supporting Information.
