# Supplementary material for: A Liquid Crystal Elastomer‐Based Unprecedented Two‐Way Shape‐Memory Aerogel
Source: Adv Sci (Weinh). 2021 Sep 26;8(22):2102674. doi: 10.1002/advs.202102674 (PMC8596101; doi:10.1002/advs.202102674)
Supplement: Supplementary file 1 — Supporting Information [file ADVS-8-2102674-s004.pdf]

© 2021 Wiley-VCH GmbH

ADVANCED SCIENCE
Open Access

Supporting Information

for *Adv. Sci.*, DOI: 10.1002/advs.202102674

Liquid Crystal Elastomer Based Unprecedented Two-Way Shape–Memory Aerogel

Meng Wang, Ying Song, Hari Krishna Bisoyi, Jian-Feng Yang, Li Liu, Hong Yang, and Quan Li**

Materials and methods.

3-Butene-1-ol, ethyl *p*-Hydrobenzoate, triphenyl phosphorus, diethyl azodicarboxylate (DEAD), sodium hydroxide, *p*-hydroxyanisole, dimethylaminopyridine (DMAP), 1,3-diisopropylcarbodiimide (DIC), hydroquinone, potassium carbonate, 11-bromo-1-undecene, and 4-hydroxybenzophenone were purchased from Aladdin Inc. and used as received without further purification.

All ^{1}H NMR spectra were recorded on a Bruker HW600 MHz spectrometer (AVANCE AV-600) or a Bruker HW300 MHz spectrometer (AVANCE AV-300), using $CDCl_3$ or DMSO-d6 as the solvent and δ 7.26 ($CDCl_3$) or δ 2.50 (DMSO) as the internal reference. Differential scanning calorimetry (DSC) thermographs were recorded on a TA Instruments Q2000 instrument (New Castle, DE) under nitrogen purge with a heating/cooling rate of 10 °C/min. Both one-dimensional (1D) WAXS and two-dimensional (2D) WAXD experiments were performed on Anton Paar SAXS point 2.0 with a TCS tage 300 temperature controller. The specimen (in a TCS sample holder) was placed in the sample chamber, which was evacuated to a pressure below 3 mbar in order to minimize the atmospheric scattering of the X-ray beam. All XRD Data were collected at a sample-to-detector distance (SDD) of 79 mm using an incident X-ray beam (50.047 keV, 0.999 mA, 1.542 Å wavelength). For each specimen, six frames of 900-s exposures were collected and averaged. Two-dimensional data was transformed to one-dimensional curves by using SAXS analysis software (Anton Paar).

Polarized optical microscopy (POM) observations of all the liquid crystalline textures of the samples were performed on an Olympus BX53P microscope equipped with a Mettler PF82HT hot stage. The images were captured using a Microvision MVDC200 digital camera with a Phenix Phmias 2008 Cs Ver2.2 software.

All mechanical property studies of the polymeric samples were performed on a dynamic mechanical analyzer (DMA Q800, TA Instrument). The SEM images were recorded on an Inspect F50 S3 field emission scanning electron microscope (FEI-SEM, America). Fourier transform infrared spectroscopy (FT-IR) spectra were recorded on a Nicolet 5700 Fourier Infrared Spectrometer (Thermo Electron Scientific Instruments Corporation, America).

Synthesis of 4-methoxyphenyl-4-(1-buteneoxy) benzoate (MBB). [1]

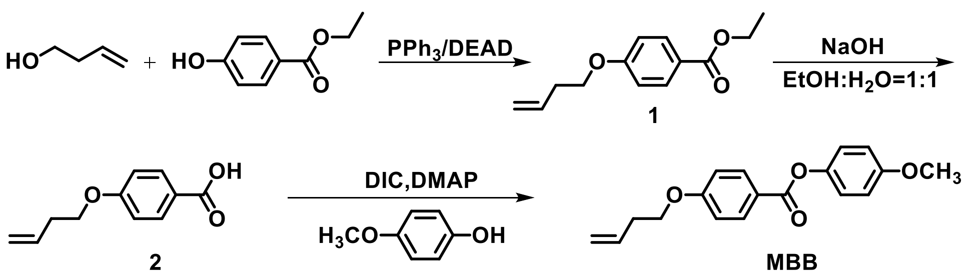

Figure S1. Synthetic route of LC monomer **MBB.**

3-Butene-1-ol (5.76 g, 80.00 mmol), ethyl *p*-hydroxybenzoate (13.28 g, 80.00 mmol) and triphenyl phosphine (21.80 g, 80.00 mmol) were added into a 100 mL three-neck flask and then 60 mL anhydrous tetrahydrofuran was added as the solvent, which was stirred by magnetons and protected by nitrogen gas. Under the condition of ice water bath, 20 mL of DEAD (13.92 g, 80.00 mmol) in tetrahydrofuran was slowly added with a constant pressure drop funnel. After dripping within 15 minutes, the ice bath device was removed and the mixture was stirred at room temperature for 24 h. The mixture was poured into other flask when it changed from yellow to white emulsion gradually, and the tetrahydrofuran solvent was removed by vacuum rotary evaporation. The obtained solids were separated by silica gel column, and the eluent was a mixture of ethyl acetate and petroleum ether at the ratio of 1:6. The collected components were decompressed and steamed, then dried for 24 h under vacuum condition to obtain 15.40 g (69.90 mmol) colorless transparent oily liquid compound **1**, with a yield of 87.36 %. The Compound **1** (15.40 g, 69.90 mmol), sodium hydroxide (5.59 g, 139.8 mmol), ethanol and water (100 mL each) were added to a 250 mL round-bottom flask, and the magnetons were added to stir. The reaction was heated to reflux for 18 h. After cooling to room temperature, the 3N hydrochloric acid aqueous solution was added to adjust the pH of the reaction solution to 2. The white solid precipitate was filtered with a Brinell funnel and rinsed with ethanol and deionized water. After vacuum drying, the white solid compound **2** (13.00 g, 67.71 mmol) were obtained with a yield of 96.87%. Compound **2** (13.00 g, 67.71 mmol), *p*-hydroxyanisole (9.23 g, 74.47 mmol) and DMAP (4.13 g, 33.85 mmol) were added into a 250 mL three-neck flask, and 140 mL CH_2Cl_2 was added as the solvent. The mixture was stirred by magnetons and protected by nitrogen gas. Then, 10 mL DIC (10.23 g, 81.24 mmol) in dichloromethane was slowly dropped into the flask with constant pressure funnel. The reaction mixture was heated to reflux at 35 °C for 15 h. The insoluble impurities were removed by funnel filtration, the solvent was removed by rotovap concentration and the resulting white solid was purified by flash column chromatography using DCM as the eluant to give the desired product MBB, which was recrystallized from ethanol to provide white crystals (16.20 g, 54.30 mmol) with a yield of

80.20%.^{1}H NMR (600 MHz, $CDCl_3$) δ 8.14 (d, *J* = 6 Hz, 2H), 7.12 (d, *J* =6 Hz, 2H), 6.98 (d, *J* = 12 Hz, 2H), 6.94 (d, *J* =12 Hz, 2H), 5.92 (m, 1H), 5.21 (d, *J* =12 Hz, 1H), 5.15(d, *J* =12 Hz, 1H), 4.10 (t, *J* = 12 Hz, 2H), 3.84 (s, 3H), 2.59 (m, 2H).

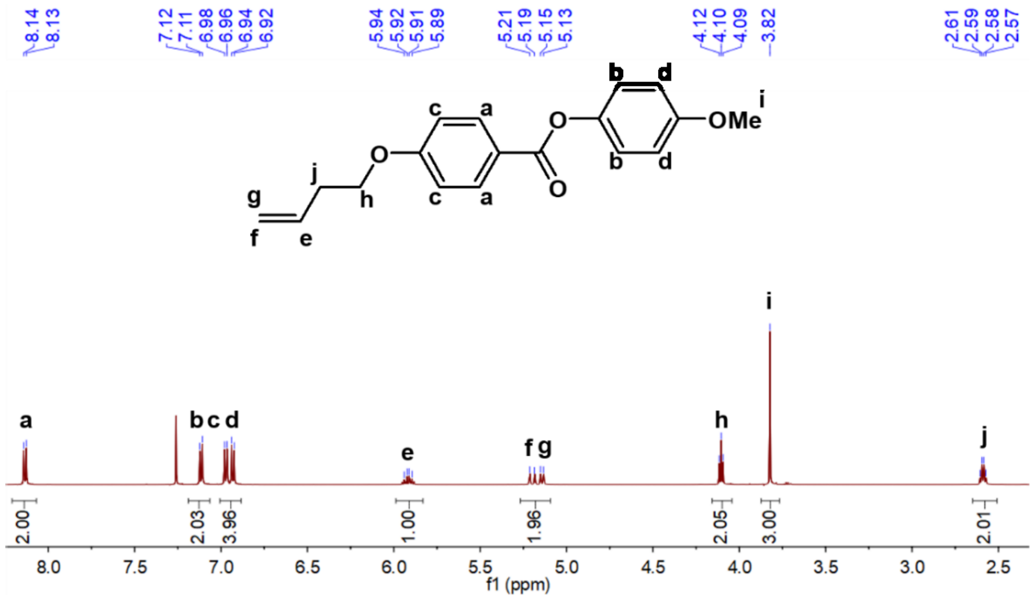

Figure S2. ^{1}H NMR spectrum of MBB.

Synthesis of 1,4-bis-undec-10-enyloxy-benzene (11UB).[1]

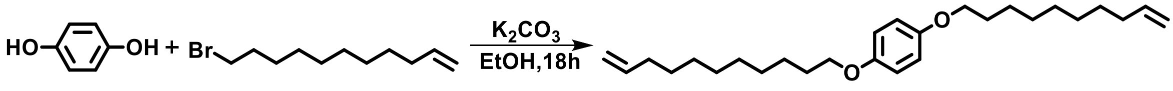

Figure S3. Synthetic route of LC crosslinker 11UB.

Hydroquinone (0.40 g, 3.64 mmol), potassium carbonate (1.75 mg, 12.73 mmol) and 10 mL anhydrous ethanol were successively added into a 50 mL three-neck flask under nitrogen protection. Then, 5 mL of 11-bromo-1-undecene (2.04 g, 8.73 mmol) ethanol solution was slowly dropped into flask with a constant pressure drip funnel. The reaction mixture was refluxed at 80 °C for 18 h. After the reaction, 60 ml of deionized water was added, and the mixture was extracted twice with 120 ml of dichloromethane. The extracted organic layer was washed with 40 mL saturated Na_2CO_3 solution and 40 mL deionized water, respectively. The organic phase obtained by separation was dried by anhydrous $MgSO_4$ and filtered. The obtained solids were separated by silica gel column, and the eluent was a mixture of ethyl acetate and petroleum ether, with a volume ratio of 1:15. The collected components were decompressed and steamed, and the white solids were recrystallized with anhydrous ethanol and dried for 24

h under vacuum condition. A total of 1.10 g (2.65mmol) white sheet crystals of 11UB were obtained, with a yield of 72.80%. ^{1}H NMR (600 MHz, $CDCl_3$) δ 6.82 (s, 4H), 5.84-5.78 (m, 2H), 5.01-4.93 (m, 4H), 3.91-3.88 (t, J=12 Hz, 4H), 2.06-2.02 (dd, J=6,12 Hz, 4H), 1.76-1.74 (m, 4H), 1.39-1.27 (m, 24H).

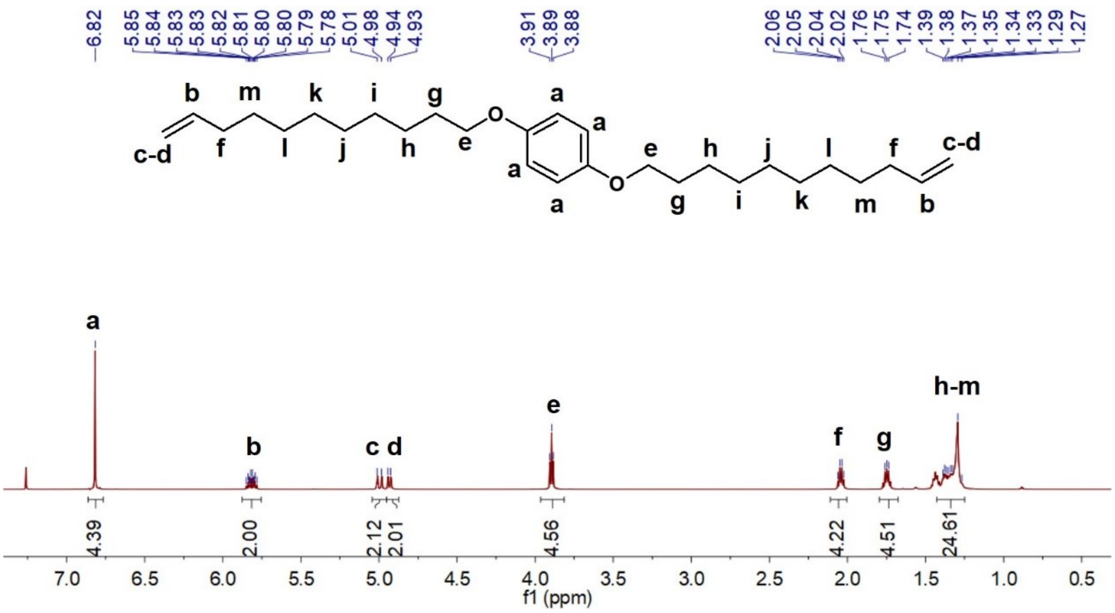

Figure S4. ^{1}H NMR spectrum of 11UB.

Synthesis of 4-(10-undecenyloxy) benzophenone (C11OBP).[2]

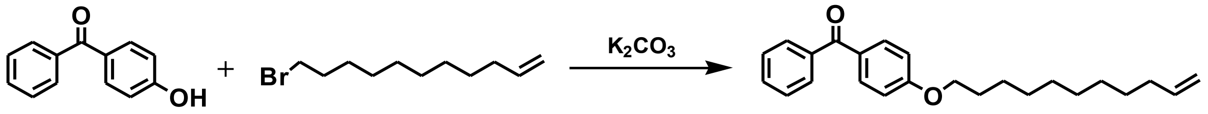

Figure S5. Synthetic route of LC crosslinker C11OBP.

4-Hydroxybenzophenone (24.77 mmol) was dissolved in 20 mL of acetone in a three necked round bottom flask and the mixture was stirred. The starting terminal-bromo-alkene compound (0.020 mol), here 11-bromo-1-undecene (20.01 mmol), was added and dissolved. After adding one equivalent of K_2CO_3 (24.69 mmol), the mixture was stirred and heated to reflux for 5 h. The mixture was subsequently allowed to cool to room temperature and 20 mL of deionized water was added. The resulting solution was extracted four times with 20 mL of diethyl ether and the combined ether phases were washed four times with 20 mL of aqueous NaOH solution (10%). The organic phase was dried with Na_2SO_4 overnight and filtered off and collected in a round bottom flask. The filter residue was washed with dried diethyl ether. The solvent was removed under reduced pressure and the raw product was obtained. For further purification, the raw product was recrystallized from methanol to yield C11OBP (3.78 g, yield: 65%).^{1}H NMR

(600 MHz, $CDCl_3$) δ 7.83 - 7.81 (d, *J* = 12 Hz, 2H), 7.76 - 7.75 (d, *J* = 6 Hz, 2H), 7.58 - 7.55 (m, 1H), 7.48-7.46 (t, *J* = 6 Hz, 2H), 6.96-6.94 (d, *J* = 12 Hz, 2H), 5.84 - 5.79 (m, 1H), 5.01-4.92 (m, 2H), 4.05 - 4.03 (t, *J* = 6 Hz, 2H), 2.06 - 2.03 (m, 2H), 1.83-1.80 (m, 2H), 1.48-1.25 (m, 12H).

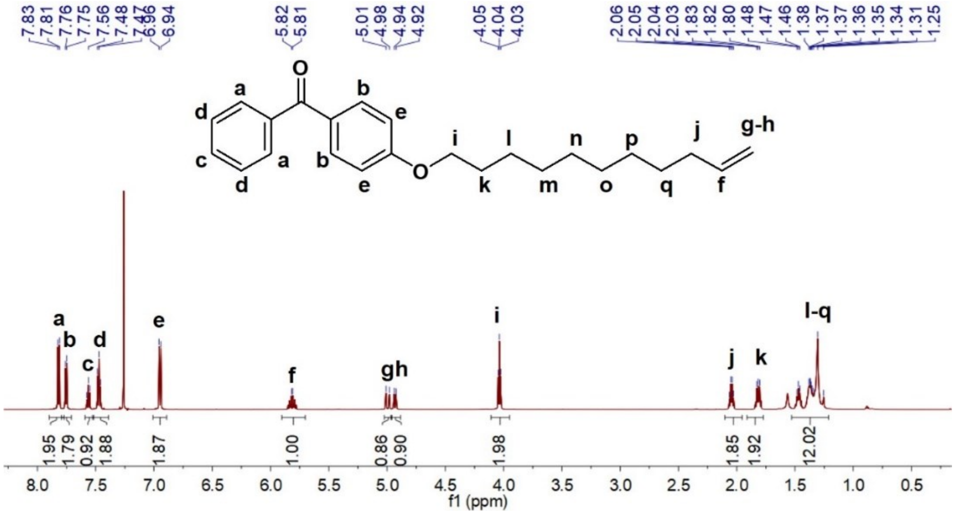

Figure S6. ^{1}H NMR spectrum of C11OBP.

Preparation of LCE-based aerogel samples.

The preparation protocol was divided into four main stages, including the synthesis of organogel, solvent exchange process, supercritical CO_2 extraction and secondary crosslinking stage.

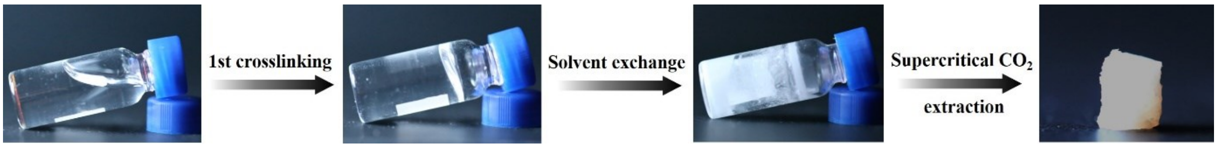

Figure S7. Preparation of the LCE-based aerogel.

(1) Fabrication of organogel. The molar ration of MBB:11UB:C11OBP was set as 65:15:5. PMMS (134.0 mg, containing 1.0 mmol S-H groups), MBB (193.7 mg, 0.65 mmol), 11UB (62.1 mg, 0.15 mmol), C11OBP (17.5 mg, 0.05 mmol), 2,2-azobisisobutyronitrile (AIBN) (4.0 mg, 0.025 mmol) and toluene (1.0 mL) were added in a 2 mL transparent glass bottle. The mixture was ultrasonicated for 10 minutes to ensure a homogeneous dispersion. Then, the hermetic bottle with reaction solution inside was heated at 60 °C in an oven for 12 h to finish the gelation of the mixture solution, and a transparent cylindrical organogel was obtained in the bottle.

(2) Solvent exchange. The obtained organogel was completely soaked in acetone for 72 h. During this process, the solvent was exchanged by fresh acetone every 24 h (three times totally)

to remove excess toluene and impurities from organogel. After solvent exchange process, the original transparent organogel became an opaque and milky white organogel.

(3) Supercritical CO_2 extraction. The organogel soaked in acetone was further treated by exchanging the acetone with liquid carbon dioxide (CO_2) using an Accudyne multivessel automated system. Firstly, an open glass bottle with wet organogel and acetone inside was placed into a sealed separation kettle. The bottom of this open glass bottle had been carefully smashed to ensure that during the following supercritical CO_2 extraction process, the supercritical CO_2 could constantly flow into the open glass bottle and adequately wash the inside entire wet gel. After turning the inside temperature to 25 °C, the separation kettle was pressurized to 10.0 MPa with CO_2 pump. Then, the acetone in the bottle was continuously exchanged by liquid CO_2 at the flow rate of 15 L/min. This solvent replacement process was repeated at least three times until no liquid flowed out from the collection vessel. After that, keeping the pressure in stable, the inside temperature of the separation kettle was raised to 50 °C. The gel was constantly washed with supercritical CO_2 at the flow rate of 10 L/min. This procedure lasted for 4-6 h to finish the whole CO_2 extraction process. Finally, the CO_2 in the separation kettle was gradually released at a degassing rate lower than 5 L/min until the pressure in the kettle was equal to the atmospheric pressure. The specimens were heated in a vacuum oven at 50 °C for 12 h to remove any solvent residue.

(4) Secondary crosslinking. The obtained pre-crosslinking LCE-based aerogel sample was carefully removed from the glass bottle and cut into a piece (9.23±0.01 mm × 3.45±0.01 mm × 2.86±0.01 mm), which was uniaxially stretched to ca. 120~130% of the original length at 50 °C. Then stretched pre-crosslinked LCE-based aerogel sample was placed under UV light (365 nm, 9000 $\mu w/cm^2$) at room temperature for 30 seconds each side to provide the fully crosslinked LCE-based aerogel sample.

In addition, for comparison experiments, we also designed and prepared three other samples with different crosslinking densities through same preparation protocols. The molar ratios of MBB: 11UB: C11OBP in the other three systems were respectively set as 70: 12.5: 5, 55: 20: 5, 45: 25: 5, while the molar ratio of thiol: ene in all the system was 1:1. There was no aerogel formation in the bottle with the molar ratio of MBB: 11UB: C11OBP = 70: 12.5: 5. The properties of these systems were measrued and listed in Table 1. Obviously, the porosity of sample 2 and sample 3 were lower than 40%, and these two systems could not be recognized as polymeric aerogel materials. The SEM images (Figure S8) also showed that the pores of sample 2 and sample 3 shrunk and collapsed, which indicated that the high crosslinking degree might reduce the porosity and increased the bulk densities of the samples. These data clearly

demonstrated that a higher degree of crosslinking was more conducive to forming a denser network, but could reduce the porosities of the aerogel materials.

Table1. Chemical compositions and physical properties of cross-sections of LCE-based aerogel samples prepared with different molar ratios of (a) **MBB**: **11UB: C11OBP** = 70: 12.5: 5; (b) **MBB**: **11UB :** **C11OBP** = 55: 20: 5; and (c) **MBB**: **11UB : C11OBP** = 45: 25: 5.

**Sample**	**[MBB]:[11UB]: [C11OBP]**	**Bulk density (g/cm^3)**	**Skeletal density (g/cm^3)**	**Porosity (%)**
sample 1	70: 12.5: 5	-	-	-
sample 2	55: 20: 5	0.596±0.012	0.9654±0.021	38.3±0.1
sample 3	45: 25: 5	0.703±0.014	0.9532±0.018	26.2±0.1

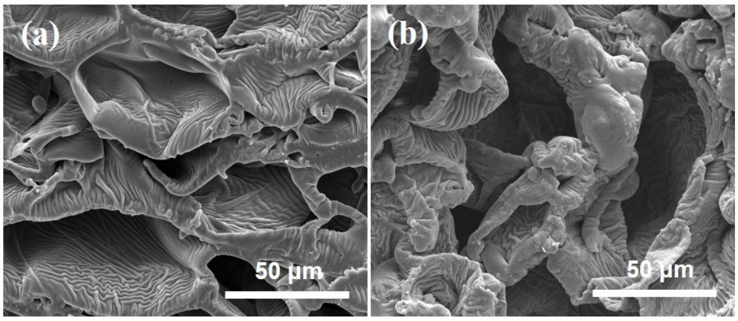

Figure S8. SEM images of cross-sections of the LCE-based aerogel samples prepared with different molar ratios of (a) MBB: 11UB : C11OBP = 55: 20: 5, (b) MBB: 11UB : C11OBP = 45: 25: 5.

To verify the successfully thiol-ene click reaction between PMMS and MBB/11UB/C11OBP, the Fourier-transform infrared spectroscopy (FT-IR) and ^{1}H NMR spectra of the pre-crosslinked LCE-based aerogel were recorded, as shown in Figure S9,S10. All monomers had an absorption peak at 1640 cm^{-1} and several sharp absorption peaks at 1450 ~ 1600 cm^{-1}, which were respectively attributed to stretching vibration of C=C of the terminal vinyl groups and skeletal vibration of phenyl groups. PMMS showed an obvious absorption around 2560 cm^{-1}, which was ascribed to the stretching vibration of sulfhydryl. After thiol-ene click polymerization reaction, the peaks at 1640 cm^{-1} and 2560 cm^{-1} completely disappeared on the FT-IR spectrum of the pre-crosslinked LCE-based aerogel, while the peaks at 1450 ~ 1600 cm^{-1} still remained. Meanwhile, after thiol-ene click polymerization reaction, the terminal olefin protons of MBB, 11UB and C11OBP originally located at 4.5 ~ 6.0 ppm all vanished completely on the ^{1}H NMR spectrum of the pro-crosslinked LCE-based aerogel, while the resonance signals of the aromatic protons still remained at 6.5 ~ 8.0 ppm. Taking account of these FT-IR and ^{1}H NMR data, it was convinced that the monomer MBB, crosslinkers 11UB

and C11OBP have been successfully grafted onto PMMS backbone *via* the thiol-ene click chemistry and there are no residual unreacted LC monomers remained in the pre-crosslinked LCE-based aerogel.

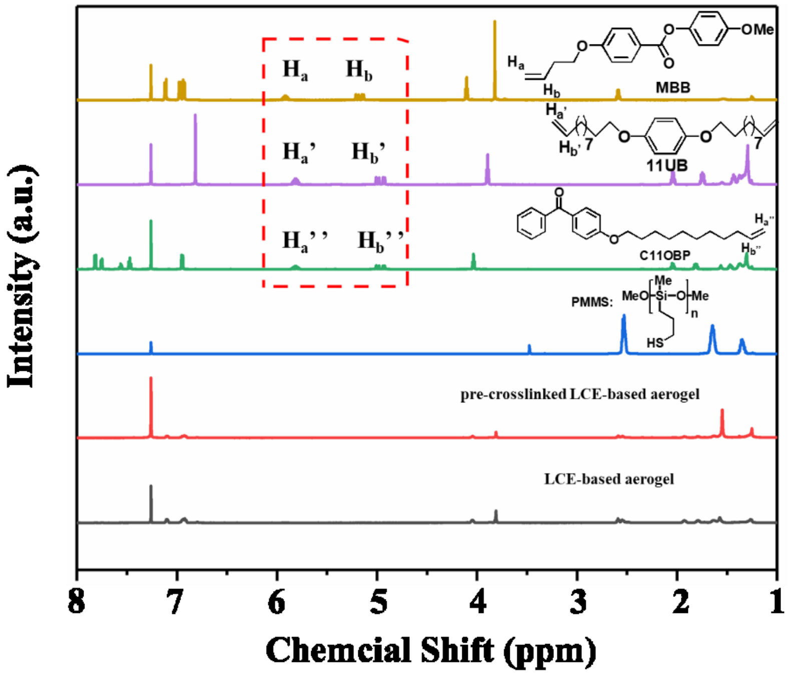

Figure S9. The ^{1}H NMR spectra of MBB, 11UB, C11OBP, PMMS, pre-crosslinked LCE-based aerogel sample and corresponding oriented LCE-based aerogel sample.

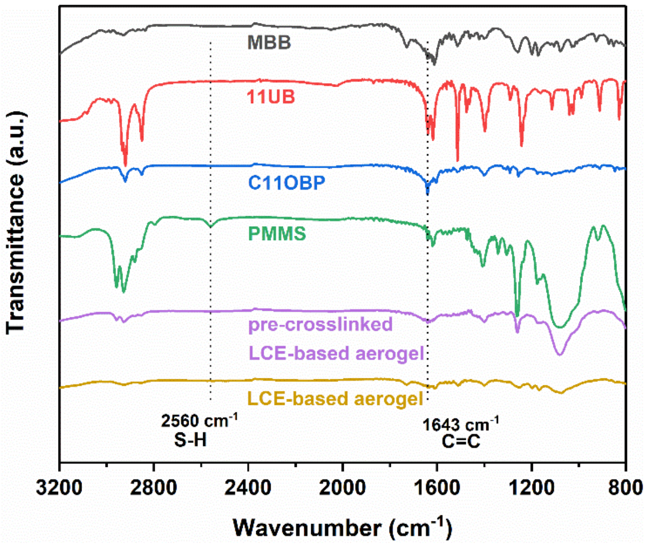

Figure S10. The FT-IR spectra of MBB, 11UB, C11OBP, PMMS, pre-crosslinked LCE-based aerogel and corresponding oriented LCE-based aerogel sample.

The thermal and mesomorphic properties of the oriented LCE-based aerogel were investigated by differential scanning calorimetry (DSC), temperature-varied wide-angle X-ray scattering (WAXS) and polarizing optical microscope (POM). As demonstrated in Figure 3a,

DSC curves of the LCE-based aerogel sample presented an enantiotropic and wide LC phase region during the heating and cooling cycles. The LCE-based aerogel had a quite low T_g around -5.4 °C and a relatively low clearing point temperature about 48.8 °C belonging to the LC-to-isotropic phase transition. During the heating and cooling cycles, the sample showed one single LC phase under POM observation, which was in good agreement with DSC result. As shown in Figure 3b, the POM image of sample showed the colorful birefringent textures at 30 °C. After heating the sample to 55 °C, the whole image turned dark entirely under POM observation (Figure S11), implying a complete LC-isotropic phase transition.

As illustrated in Figure 3c and S12, one-dimensional (1D) WAXS patterns of the LCE-based aerogel presented one diffuse peak in wide-angle region and a weak peak in low-angle region during the heating and cooling processes. When the temperature rose to above T_{iso}, the peak in wide-angle region became flat and the peak in low-angle region disappeared totally, implying a nematic phase of the LCE-based aerogel. Correspondingly, as shown in two-dimensional (2D) WAXS patterns of the LCE-based aerogel sample (Figure 3d), at 30 °C, a pair of small arcs in a low-angle area were gathered on the equator, and a pair of narrow crescents in a wide-angle area were gathered on the meridian, which proved that the mesogens were well oriented along the stretching direction. In view of the DSC, POM and WAXS results, the LCE-based aerogel possesses a LC phase in the temperature region of -5.4 ~ 48.8 °C and the mesogens were well oriented along the stretching direction.

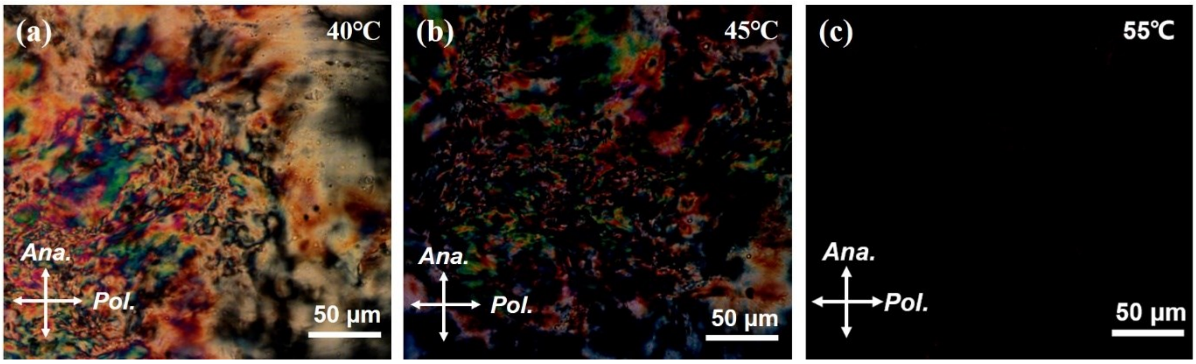

Figure S11. POM images of LCE-based aerogel sample recorded at (a) 40 °C, (b) 45 °C and (c) 55 °C.

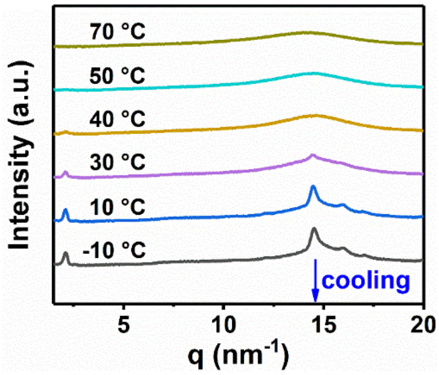

Figure S12.1D-WAXS patterns of LCE-based aerogel sample on cooling.

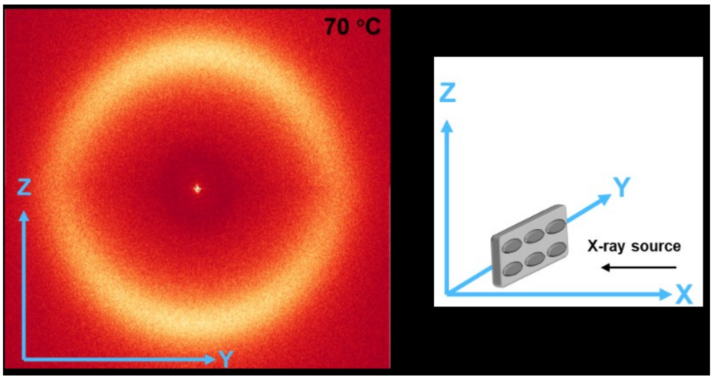

Figure S13. 2D-WAXS pattern of LCE-based aerogel sample at 70 °C.

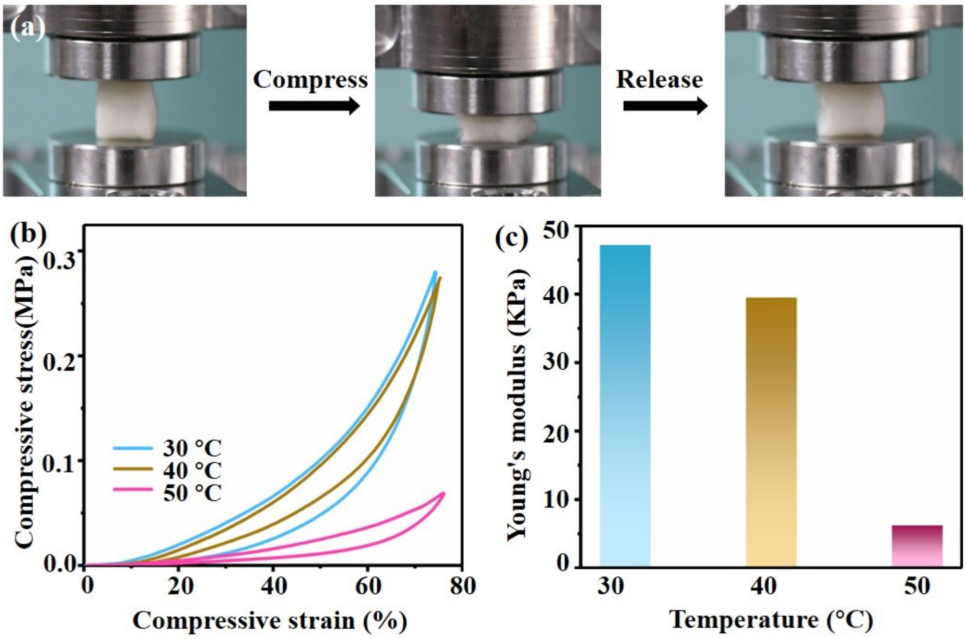

Figure S14. (a) Compression and recovery test of the LCE-based aerogel sample perpendicular to the stretching direction. (b) The cyclic compression-relaxation curves and (c) the corresponding Young's modulus of the LCE-based aerogel sample at different temperatures.

Statistical Analysis.

The experimental results for ^{1}H NMR spectra, FT-IR spectra, DSC curves, SEM images,

POM images, 1D-WAXS patterns, 2D-WAXS patterns, DMA curves and digital pictures were shown as raw data and no data pre-processing and statistical analysis have been used. All the BET, dimension and density data were expressed as mean ± standard error. An error bar of one standard deviation is added in the data wherever applicable. All the data were plotted with OriginPro 2017 (OriginLab Corp.).

References

[1] Li C, Liu Y, Lo C, et al. *Soft Matter* **2011**, *7*, 7511.
[2] Nordendorf G, Schafforz S L, Käkel E B, et al. *Phys. Chem. Chem. Phys.* 2**020**, *22*, 1774.

Supporting videos:

Movie S1. The video S1 was the 75% compression test along the stretching direction of LCE-based aerogel.
Movie S2. The video S2 was the 75% compression test perpendicular to the stretching direction of LCE-based aerogel.
Movie S3. The video S3 was a thermal-induced two-way shape memory behavior of the LCE-based aerogel.
